# NTRC and Thioredoxin f Overexpression Differentially Induces Starch Accumulation in Tobacco Leaves

**DOI:** 10.3390/plants8120543

**Published:** 2019-11-26

**Authors:** María Ancín, Luis Larraya, Alicia Fernández-San Millán, Jon Veramendi, Tessa Burch-Smith, Inmaculada Farran

**Affiliations:** 1 Institute for Multidisciplinary Research in Applied Biology, UPNA, 31006 Pamplona, Spain; maria.ancin@unavarra.es (M.A.); luis.larraya@unavarra.es (L.L.); alicia.fernandez@unavarra.es (A.F.-S.M.); jon@unavarra.es (J.V.); 2 Department of Biochemistry and Cellular and Molecular Biology, University of Tennessee, Knoxville, TN 37996, USA; tburchsm@utk.edu

**Keywords:** starch metabolism, thioredoxin, NTRC, redox regulation, chloroplast, VIGS

## Abstract

Thioredoxin (Trx) f and NADPH-dependent Trx reductase C (NTRC) have both been proposed as major redox regulators of starch metabolism in chloroplasts. However, little is known regarding the specific role of each protein in this complex mechanism. To shed light on this point, tobacco plants that were genetically engineered to overexpress the NTRC protein from the chloroplast genome were obtained and compared to previously generated Trx f-overexpressing transplastomic plants. Likewise, we investigated the impact of NTRC and Trx f deficiency on starch metabolism by generating *Nicotiana benthamiana* plants that were silenced for each gene. Our results demonstrated that NTRC overexpression induced enhanced starch accumulation in tobacco leaves, as occurred with Trx f. However, only Trx f silencing leads to a significant decrease in the leaf starch content. Quantitative analysis of enzyme activities related to starch synthesis and degradation were determined in all of the genotypes. Zymographic analyses were additionally performed to compare the amylolytic enzyme profiles of both transplastomic tobacco plants. Our findings indicated that NTRC overexpression promotes the accumulation of transitory leaf starch as a consequence of a diminished starch turnover during the dark period, which seems to be related to a significant reductive activation of ADP-glucose pyrophosphorylase and/or a deactivation of a putative debranching enzyme. On the other hand, increased starch content in Trx f-overexpressing plants was connected to an increase in the capacity of soluble starch synthases during the light period. Taken together, these results suggest that NTRC and the ferredoxin/Trx system play distinct roles in starch turnover.

## 1. Introduction

Post-translational redox modifications play a major role in different cell processes, with thioredoxins (Trxs) being among the protagonists in this regulatory mechanism [1]. The Trxs modulate target protein activities via a thiol-disulfide exchange mechanism that involves the two redox-active Cys residues of the canonical WC(G/P)PC active site [2]. In plants, the Trx system is particularly complex, with a great number of Trx isoforms [3] and more than 400 potential Trx targets [4]. The first experimental evidence of redox regulation and signalling in a biological system was reported in plant chloroplasts [5]. In illuminated chloroplasts, ferredoxin (Fd) and Fd-Trx reductase (FTR) are responsible for feeding the electrons that were generated in the photosynthetic electron transport chain into the regulatory Trx system. In the dark, or in non-photosynthetic plastids, the NADPH that is generated from the oxidative pentose phosphate pathway (OPPP) is the source of reducing power for Fd reduction via Fd–NADPH reductase (FNR) [6]. Plastid Trxs act as transmitters of the redox signal by transferring electrons from Fd to downstream target enzymes. Five types of classical Trxs (MW about 10–12 kDa) have been reported for plastids (Trx f, m, x, y, z) [7,8], as well as “Trx-like” proteins with non-canonical active sites, or “atypical Trxs”, in which the Trx motifs are associated with other domains [9].

C-type NADPH-dependent Trx reductase (NTRC), which was first identified in rice [10], has been classified as an atypical Trx [9], but also as a Trx reductase [11]. Its Trx domain is an extension (at the C-terminus) of the NADPH-Trx reductase (NTR) domain. NTRC is only found in photosynthetic organisms, such as plants, algae, and certain cyanobacteria [10,12], both in chloroplasts and non-green plastids [13]. It has been described as a bifunctional enzyme that reduces target proteins while using NADPH as an electron donor [14,15,16]. The homodimer of NTRC is reported to be catalytically active [17]. The discovery of NTRC thus unveiled the existence of a redox regulatory system in chloroplasts, other than that provided by Fd/Trx, which operates during both light and dark periods while using the NADPH that was generated from photosynthesis and OPPP, respectively. Some authors suggest that NTRC becomes a key reducing system during the night, when the level of reduced Fd is low [12,16].

Starch, which is composed of amylose and amylopectin, is the most abundant storage carbohydrate in plants. Transitory starch is considered to be a carbohydrate reservoir that buffers the diurnal changes in the supply of photoassimilates. ADP-glucose pyrophosphorylase (AGPase), which catalyzes the first committed step of starch synthesis in higher plants, is considered to be a key enzyme in this process, controlling the flux of carbon into starch [18]. Additionally, an alternative model that considers the transport of ADP-glucose (generated in the cytosol through sucrose synthase activity) into the chloroplast has also been proposed [19,20,21]. Three major classes of enzymes act simultaneously in starch synthesis: starch synthases (SSs), branching enzymes (BEs), and debranching enzymes (DBEs). Soluble SSs (four isoforms in higher plants) are believed to be primarily responsible for amylopectin synthesis, catalyzing the formation of a polymer of α-1,4-glycosidic bonds while using ADP-glucose. BEs create branches from existing chains via glucanotransferase reactions, and DBEs (ISA1 and ISA2) hydrolyze some of the branches again during the synthesis of amylopectin [22]. For starch degradation, starch granule surface solubilization is required to provide hydrolases access to the glucan chains. This process is dependent on the reversible phosphorylation and dephosphorylation of glucans, which are completed by glucan, water dikinases (GWDs), phosphoglucan phosphatases (PWDs), and a glucan-binding phosphatase (SEX4) [23]. The complete degradation of starch is achieved by endo-acting α-amylases (AMYs) and exo-acting β-amylases (BAMs) enzymes, along with DBEs (ISA3 and LDA) for hydrolysis of the branch points [23].

Starch metabolism is extraordinarily well tuned due to the presence of very sophisticated regulatory mechanisms. Post-translational modifications, including redox regulation, are the main way by which the activity of enzymes that are involved in transient starch metabolism are regulated [24,25]. In higher plants, AGPase was described as a heterotetramer consisting of two large (AGPS) and two small (AGPB) subunits. In vitro experiments demonstrated that Trxs mediate its activation by the reduction of a disulfide bridge between the two small subunits [26,27]. Arabidopsis *trxf* and *ntrc* mutants, which showed a decrease both in starch content and in AGPase redox activation, verified the in vivo relevance of these findings [28,29,30,31,32]. From these works, it is proposed that Trx f is a key enzyme that is involved in light-dependent activation of AGPase, while NTRC has been identified as AGPase regulator in the dark in response to sugars. However, the impact of the redox regulation of AGPase in vivo and its role in starch synthesis have fallen under discussion [19,33,34,35]. Other starch biosynthetic enzymes, like ISA1/ISA2, SS1, and SS3, have been reported to be susceptible to reducing agents [36]. However, only SS1 has been confirmed as a redox-regulated enzyme in vitro, being mainly activated by Trx f, but also by Trx m and NTRC [37].

In addition to starch synthesis, enzymes that are involved in starch degradation have been shown to be under redox control [24,36]. Among them, chloroplast localized AMY3 could be activated by reduced Trxs in Arabidopsis, with Trx f being the most effective [38]. The same applies to BAM1, which NTRC could also partially activate [39,40]. The enzyme GWD, which catalyzes the phosphorylation of starch, and SEX4, which is required for glucan desphosphorylation, can also be reduced and activated by Trxs in vitro, with Trx f being the more efficient activator [41,42,43]. Nevertheless, Arabidopsis *gwd* mutant plants expressing a redox-insensitive GWD displayed normal starch turnover [44]. According to the widely accepted model for the night-active starch degradation pathway [23,45], the reductive activation of starch degradative enzymes by Trxs (during the day) may seem to be contradictory. However, there is considerable evidence supporting that starch degradation also takes place in such conditions, being mainly associated with certain stresses or guard cell opening [39,46], indicating the existence of starch degradation in leaves during the day and, probably, its biosynthesis at night, as previously proposed [19,33,47]. Despite this fact, NTRC could also provide the reducing power to redox-activate starch turnover during the night.

A combination of in vitro and in vivo evidence indicates that Trx f and NTRC both clearly modulate transitory starch metabolism in leaves, although a distinguishable role for each Trx type in vivo is unclear. Previous results showed that Trx f overexpression from the chloroplast genome promotes starch accumulation in tobacco leaves [48]. Therefore, plastid transformation was used to generate NTRC overexpressing tobacco plants, so as to examine whether NTRC and Trx f perform discernible and specific functions on the regulation of starch metabolism. Moreover, *Nicotiana benthamiana* plants with silenced *ntrc* or *Trxf* genes were generated as the controls for enzymatic analyses. Our results help in gaining insights into Trx specificity in vivo, showing that NTRC overexpression promotes the accumulation of transitory leaf starch by diminishing starch turnover during the dark period, while starch synthase activation appears to be the main determinant of starch accumulation in Trx f transgenic plants.

## 2. Results

### 2.1. Generation of Transplastomic Tobacco Plants Overexpressing a Fully Functional NTRC

**Figure 1:**
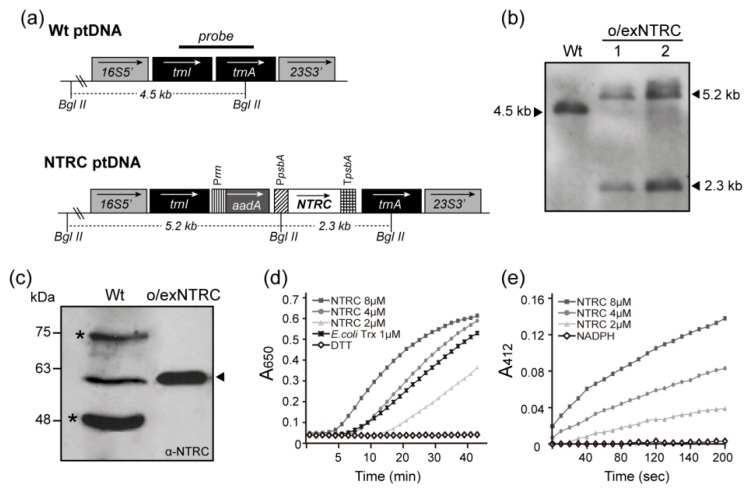
The integration, overexpression, and functionality of the NADPH-dependent Trx reductase (NTRC) protein, and homoplasmy confirmation of transplastomic tobacco plants (T_1_ generation). (**a**) Map of the wild-type (Wt) and transformed NTRC plastid genomes. The *ntrc* gene, driven by the psbA promoter and terminator, was cloned into the intergenic region between trnI and trnA. The arrows in the boxes show the direction of transcription. The probe for the Southern blot is shown over the corresponding sequence. The sizes of the predicted bands after DNA digestion with BglII are indicated. 16S5′, trnI, trnA, 23S3′: original sequences of the chloroplast genome; aadA: aminoglycoside 3′-adenylytransferase gene; Prrn: 16SrRNA promoter and 5′-untranslated region; PpsbA: psbA promoter and 5′-untranslated region; and, TpsbA: terminator region of the psbA gene. (**b**) Southern blot analysis of Wt and o/exNTRC plants. The sizes of the bands after DNA digestion with BglII are indicated. (**c**) Immunoblot analysis of total protein extracts from fully-expanded leaves of seven week-old plants. Protein loading for o/exNTRC (1 µg) and Wt (50 µg) extracts was adjusted to make NTRC bands visible in the blot. A specific primary anti-NTRC antibody [10] at 1:750 dilution was used. The arrowhead indicates the NTRC band. Asterisks indicate non-specific bands. (**d**) Dithiothreitol (DTT)-dependent insulin reduction assay of NTRC was performed in a reaction mixture containing 2, 4, and 8 µM of purified NTRC, supplemented with 0.5 mM DTT. Negative control runs were performed in the absence of NTRC (line DTT in graph). Trx from E. coli (1 µM) was used as a positive control. (**e**) NADPH-dependent reduction of DTNB was assayed at room temperature in a buffer containing 2, 4, and 8 µM of NTRC supplemented with 150 µM NADPH. Negative controls runs were performed in the absence of NTRC (line NADPH in graph).

The mature Arabidopsis *ntrc* coding sequence was placed under the control of the plastid *psbA* promoter and terminator and then inserted, together with the selectable spectinomycin resistance gene *aadA,* into the tobacco plastid genome between the *trnI* and *trnA* genes for chloroplast expression (Figure 1a). Southern blotting verified site-specific integration and then confirmed the homoplasmy of the regenerated spectinomycin-resistant plants. The flanking region probe (Figure 1a) identified a 4.5 kb hybridizing fragment in Wt plants, and then recognized two fragments (5.2 and 2.3 kb) in the NTRC-overexpressing lines (Figure 1a,b), which are hereafter referred to as o/exNTRC. The absence of 4.5 kb bands in o/exNTRC plants indicated homoplasmy.

Immunodetection confirmed NTRC overexpression, with the overexpressed and the endogenous proteins showing similar mobility (Figure 1c). In the Wt plants, the NTRC band was visible in the blot when 50 µg of total protein were loaded, whereas only 1 µg of total protein was sufficient for the o/exNTRC plants. Densitometric analysis of several immunoblots showed the NTRC in the transplastomic plants to be some 200 times more abundant than endogenous NTRC in the Wt plants. Moreover, the affinity-purified NTRC from tobacco chloroplasts catalysed both the reduction of insulin in the presence of dithiothreitol (DTT) (Figure 1d) and the reduction of DNTB in the presence of NADPH (Figure 1e) in a concentration-dependent manner. Thus, the NTRC that was overexpressed in tobacco chloroplasts was fully functional and it showed both Trx and NTR activities.

**Figure 2:**
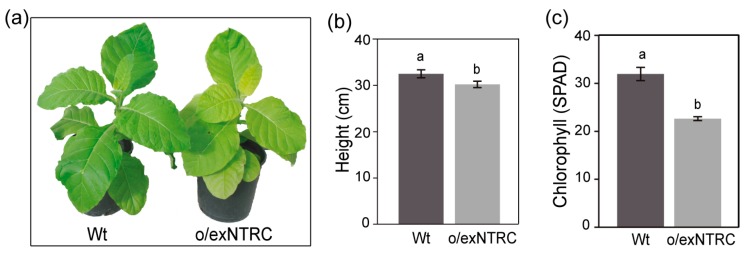
Phenotypic characterization of the T_1_ generation of NTRC-overexpressing homoplasmic plants. (**a**) Wt and o/exNTRC plants were grown in phytotron for seven weeks under standard conditions (150 μmol photons m^−2^ s^−1^, 16-h photoperiod and 28 °C). Mean stem height (**b**) and chlorophyll content (**c**) (SPAD value) was measured. Values ± SE were obtained from 15 plants per line. Different letters represent significant differences between lines (*p* < 0.05, ANOVA).

Phenotypically, o/exNTRC plants were slightly, but significantly, smaller than Wt plants (Figure 2a,b). Moreover, their leaf chlorophyll content was approximately 30% lower than that of the Wt plants (Figure 2c), as consistent with the pale-green color of o/exNTRC plants (Figure 2a). However, these differences disappeared by the adult stage (Table S1).

### 2.2. Production of Nicotiana Benthamiana Plants with Reduced Expression of NTRC or Trx f

**Figure 3:**
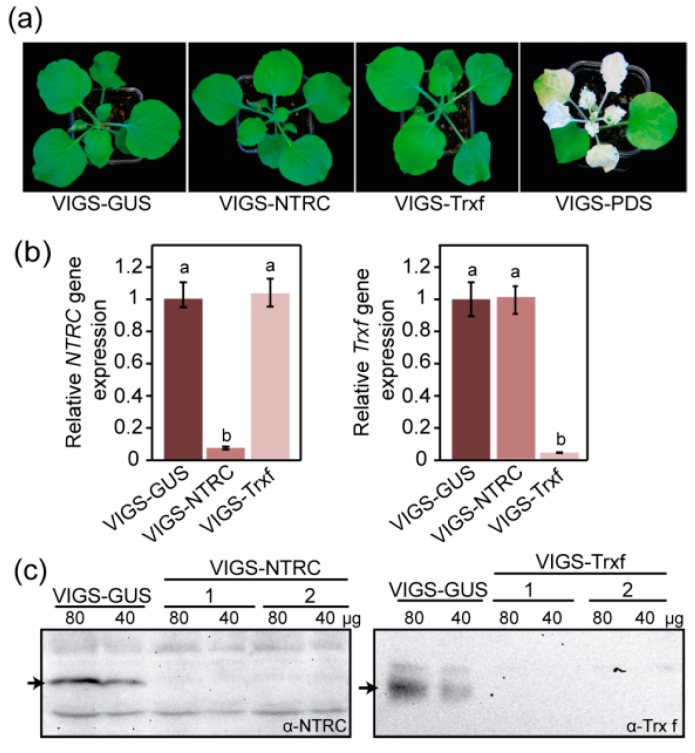
NTRC and Trx f silencing in *N. benthamiana* plants. (**a**) Representative photographs of virus induced gene silencing-NTRC (VIGS-NTRC) and VIGS-Trxf plants taken three weeks after infiltration. The negative control VIGS-GUS and the positive control VIGS-PDS were also shown. (**b**) Suppression rate of *ntrc* and *Trxf* genes in VIGS plants analyzed by RT-qPCR three weeks after infiltration. The relative transcript levels of *ntrc* and *Trxf* in VIGS-NTRC and VIGS-Trxf plants were expressed relative to that of the negative control (VIGS-GUS), previously normalized to 1. The data represent means ± SE of six biological replicates. Statistical significance as compared to control plants is indicated by different letters (*p* < 0.05, ANOVA). (**c**) Immunoblot analysis of NTRC and Trx f protein level in silenced plants when compared to VIGS-GUS plants. 40 and 80 µg of total protein extracts from each line were separated by electrophoresis on 10% and 15% SDS-PAGE for NTRC and Trx f, respectively. Protein detection was performed with a primary antibody, anti-NTRC [10], or anti-Trx f [51], at 1:750 or 1:5000 dilution, respectively. Arrow refers to NTRC in the left blot and to Trx f in the right blot.

Tobacco rattle virus-based virus induced gene silencing (VIGS) was applied to efficiently silence the *ntrc* or *Trxf* genes in *N. benthamiana* because the potent antiviral machinery of tobacco limits the efficacy of VIGS in this species [49]. To construct the gene-silencing vectors, conserved regions of *ntrc* and *Trxf* cDNA were individually cloned into the pTRV2 (TRV RNA2) vector [50]. Co-infiltration of Agrobacterium cultures carrying pTRV1 (TRV RNA1) or pTRV2-Trxf or –NTRC derivatives into *N. benthamiana* seedlings initiated VIGS. VIGS-NTRC and VIGS-Trxf plants showed no apparent phenotypes three weeks after infiltration and they were indistinguishable from non-silencing control plants (VIGS-GUS) under the assayed growing conditions (Figure 3a). By contrast, VIGS-PDS plants, used as positive controls for gene silencing, efficiently induced the photobleaching of *N. benthamiana* leaves (Figure 3a). The RT-qPCR analysis revealed that *ntrc* and *Trxf* gene expression was efficiently and specifically reduced in each VIGS-silenced line (Figure 3b). Hence, VIGS-NTRC plants exhibited a reduction of 92% in the *ntrc* expression without any effect on *Trxf* gene expression. Similarly, VIGS-Trxf plants specifically reduced *Trxf* gene expression by approximately 96% when compared to VIGS-GUS plants, while *ntrc* expression remains unchanged (Figure 3b). The high silencing efficiency that was achieved in these plants was also confirmed at protein level by western blot (Figure 3c). The bands corresponding to those of NTRC and Trx f proteins were significantly reduced in the total protein extracts of VIGS-NTRC and VIGS-Trxf plants, respectively (Figure 3c).

### 2.3. Transient Starch Accumulation Pattern in Leaves of Plants with Altered NTRC or Trx f Levels

**Figure 4:**
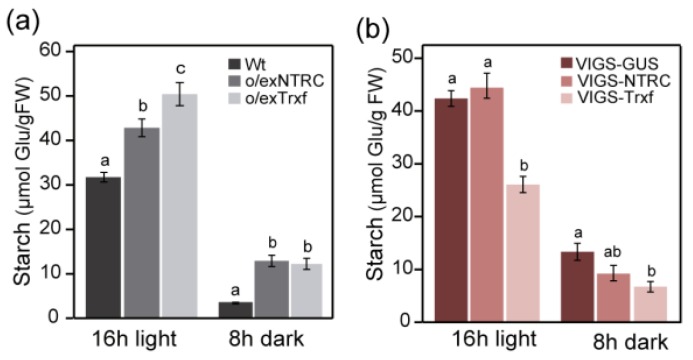
Transient starch accumulation in plants with altered NTRC or Trx f levels. (**a**) Starch content (µmol Glu/gFW) in fully-expanded leaves from seven-week-old o/exNTRC and o/exTrxf homoplasmic plants (T_1_ generation) grown in phytotron under 150 µmol m^−2^ s^−1^ after 16 h-light or 8 h-dark periods. (**b**) Starch content from mature leaves of phytotron-grown silenced plants harvested 21 days after infiltration at the end of the light (16 h; 80 µmol m^−2^ s^−1^) and dark (8 h) periods. Results are the mean ± SE of six measurements from individual plants. Different letters above the bars indicate significant differences among lines for each specific harvesting period (*p* < 0.05, ANOVA).

We further investigated the leaf starch content in plants with increased levels of NTRC (o/exNTRC) to gather additional evidence regarding the role of plastid Trxs in starch metabolism. For comparison, we included the Trx f overexpressing line (o/exTrxf), which is characterized by an enhanced starch accumulation in leaves [48]. Our results showed an increased leaf starch amount in both o/exNTRC and o/exTrxf plants as compared to Wt at the end of the day (16 h light) by about 35% and 60%, respectively (Figure 4a). The same, but even more pronounced, trend was shown in these plants at the end of the night period (8 h dark), with a similar increase in both of the genotypes that almost tripled the starch content over the Wt plants (Figure 4a). Curiously, when the starch content was analyzed in leaves of plants with reduced levels of NTRC or Trx f, only VIGS-Trxf plants showed a significant decrease, whereas the VIGS-NTRC plants achieved similar starch quantities when compared to the VIGS-GUS control plants (Figure 4b). As expected, less starch was produced at night in both genotypes.

### 2.4. Comparative Analysis of Enzymes Related to Starch Synthesis

**Figure 5:**
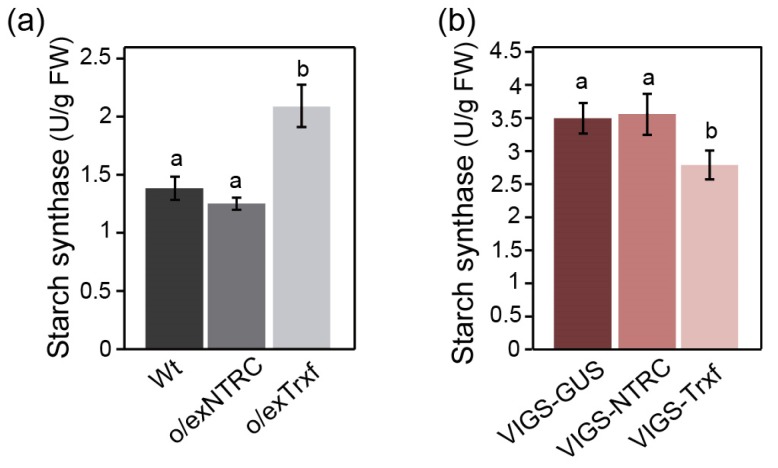
Starch synthase activity in leaves of plants overexpressing or silencing NTRC or Trx f. Protein extracts from leaves of homoplasmic tobacco overexpressing plants (T_1_ generation) (**a**) and *N. benthamiana* silenced plants (**b**) harvested after 8 h of illumination were used to analyze the soluble SS activity, as described in “Materials and Methods”. Each value is the mean ± SE of six measurements from individual plants. Different letters above the bars indicate significant differences (*p* < 0.05, ANOVA).

The activities of two well-known redox regulated enzymes, SS and AGPase [24], were analyzed in order to investigate whether o/exNTRC and o/exTrxf plants show higher starch synthesis capacity. Interestingly, o/exTrxf plants displayed a clear increase on SS activity, while the o/exNTRC plants showed no differences when compared to Wt plants (Figure 5a). Accordingly, when the silenced plants were analyzed, the VIGS-Trxf plants showed significantly lower SS activity as compared to the VIGS-GUS control plants (Figure 5b), while no differences were found in the VIGS-NTRC plants.

The levels of mRNA transcripts of two putative *N. tabacum* soluble SS protein-coding sequences were analyzed using RT-qPCR in order to investigate if o/exNTRC and o/exTrxf plants show a higher starch synthesis capacity (Figure S1). It must be mentioned that only two soluble SS orthologous genes from *N. tabacum* are well identified (*SS1* and *SS3*). Given that the transcript levels of these two genes remained unchanged in the o/exTrxf genotype (Figure S1), a specific posttranslational regulation of this enzyme by Trx f can be suggested.

**Figure 6:**
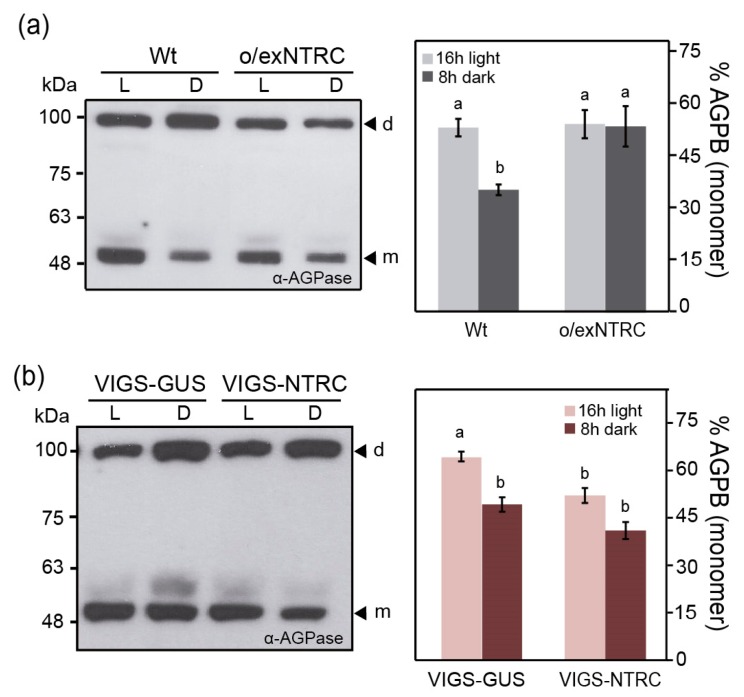
Redox activation of ADP-glucose pyrophosphorylase (AGPase) in plants with altered levels of NTRC. (**a**) Leaves of Wt and o/exNTRC homoplasmic plants *(*T_1_ generation) were sampled at the end of the light (L; 150 µmol m^−2^ s^−1^) and dark (D) periods. (**b**) Leaves of non-silencing control plants (VIGS-GUS) and VIGS-NTRC plants were harvested at the end of the light (L; 80 µmol m^−2^ s^−1^) and dark (D) periods. A representative non-reducing western blot of AGPB (AGPase small subunit) is shown in each case. m: monomer; d: dimer. AGPB monomerization is presented as the percentage of the 50-kDa monomer relative to the total amount of AGPB. Each value is the mean ± SE of four individual plants. Different letters above bars represent significant differences (*p* < 0.05, ANOVA).

Regarding AGPase, we previously demonstrated that its redox state, as a proxy of its in vivo activity, was not affected in o/exTrxf plants [48]. In the same way, VIGS-Trxf plants were analysed in this study, and no differences on the AGPase redox state as compared to control plants were found (Figure S2). The degree of AGPB monomerization was analysed by western blotting under non-reducing conditions to assess the AGPase redox activation in o/exNTRC plants. Densitometric quantification of the oxidized (~100 kDa, dimer) and reduced (~50 kDa, monomer) forms of AGPB revealed no differences in AGPB reduction between o/exNTRC and Wt plants at the end of the light phase (Figure 6a). In contrast, the o/exNTRC plants showed an increase in the AGPB monomerization when compared to Wt at the end of the dark period (Figure 6a). A surprising increase in monomerization of 2-Cys peroxiredoxin (Prx), a well-known NTRC target [52], during the dark period also occurred in o/exNTRC plants (Figure S3), which suggests that NTRC is mainly operating as a reducer in tobacco o/exNTRC plants during the night. When the VIGS-NTRC plants were analyzed, a reduction in the AGPB monomerization was shown under both light and dark conditions as compared with VIGS-GUS control plants (Figure 6b), although thedifferences were only statistically significant at the end of the light period.

Overall, our results point to a putative specific regulation of SS by Trx f and suggest that the starch-related phenotype of o/exNTRC plants might be a consequence of altered night-time starch metabolism due to the higher reduction of AGPase during this period.

### 2.5. Comparative Analysis of Enzymes Related to Starch Degradation

**Figure 7:**
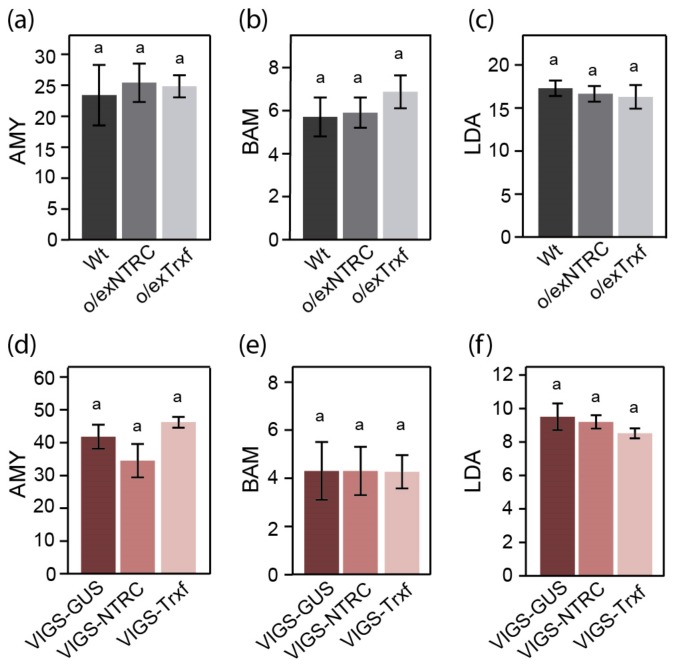
Starch-degrading enzyme activities in leaves of Wt and transgenic plants overexpressing (**a**–**c**) or silencing (**d**–**f**) NTRC or Trx f. Measurements were performed in leaves harvested after 4 h of darkness. Data are shown as units.mg^−1^ FW calculated according to Ceralpha^®^, Betamyl-3^®^ and PullG6 methods (Megazyme). Data are given as means ± SE of six extracts, each made from an individual plant. Statistical significance is indicated by different letters above the bars (*p* < 0.05, ANOVA).

We also analyzed whether the higher starch content that was observed in NTRC- or Trx f-overexpressing tobacco leaves could be related to a downregulation of enzymes that are involved in starch degradation. To this end, the activities of AMY, BAM, and LDA were measured in leaves that were harvested after 4 h of darkness from plants overexpressing NTRC or Trx f. Our results showed no differences in any of these enzyme activities among Wt, o/exNTRC and o/exTrxf plants (Figure 7a–c). Equally, no differences between lines were found in AMY, BAM, and LDA activities when plants with reduced levels of NTRC or Trx f were analyzed (Figure 7d–f), suggesting that neither NTRC nor Trx f seem to alter the overall activity of these amylolytic enzymes in tobacco leaves, at least in the assayed growing conditions. In addition, these results indicate that the increased transitory starch accumulation in o/exNTRC or o/exTrxf leaves does not occur at the expense of the deactivation of these starch-degrading enzymes.

### 2.6. Qualitative Study of Redox Sensitive Amylolytic Enzymes in Tobacco Plants

**Figure 8:**
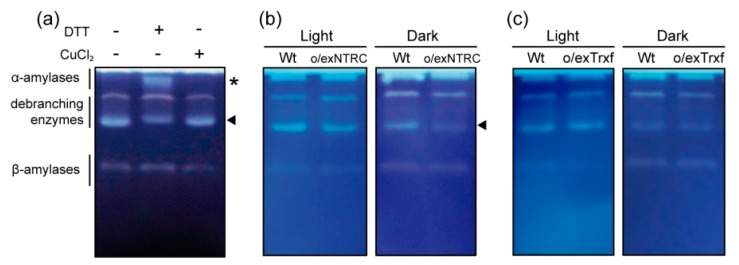
Analysis of amylolytic enzymes using native PAGE in Wt and NTRC- or Trx f-overexpressing homoplasmic tobacco leaves (T_1_ generation). Soluble proteins were extracted from leaves harvested after 8 h light or 4 h dark periods and separated (35 µg) in native polyacrylamide gels containing 0.2% potato amylopectin. After electrophoresis, the gels were incubated overnight and then stained with iodine solution to reveal pale bands where the amylopectin had been hydrolyzed. Putative activities of α-amylases, β-amylases and debranching enzymes were identified according to [53]. Asterisks indicate enzymes activated by reduction; arrowheads indicate enzymes deactivated by reduction. (**a**) Redox mediated changes in starch amylolytic activities. Extracted proteins were treated with 40 mM DTT or 200 µM CuCl_2_ for 2 h prior to gel loading. (**b**) Differences between Wt and o/exNTRC plants in light and dark conditions. (**c**) Differences between Wt and o/exTrxf plants in light and dark conditions. Representative panels from three independent experiments were shown for each line.

Zymographic analyses were performed in order to determine the amylolytic enzyme profiles of tobacco extracts. We first analyzed the redox sensitivity of starch-degrading enzymes in Wt tobacco plants by incubating the protein samples in the presence of the reducing reagent DTT or the oxidizing reagent CuCl_2_, followed by zymogram analysis. Several starch-modifying activities were observed in tobacco leaf extracts under these conditions, including AMYs, BAMs, and DBEs (Figure 8a), which were previously identified [53]. We found that the activity of a band corresponding to a putative DBE was less noticeable when incubated with DTT (Figure 8a, as indicated by an arrowhead). In contrast, a putative AMY was shown to be active only under reducing conditions (Figure 8a, indicated by an asterisk). However, oxidant conditions did not appear to modify the activity of the amylolytic enzyme profile (Figure 8a). Based on this qualitative assay, we zymographically analyzed the activity of starch degradative enzymes in o/exNTRC and o/exTrxf extracts when compared to Wt. Our results revealed a similar amylolytic enzyme pattern between the o/exNTRC and Wt plants in light conditions, while a slight decrease in the activity of a putative DBE was shown in o/exNTRC extracts in the dark (Figure 8b, arrowhead). Conversely, there was no difference in the activity of the starch-degrading enzymes between Wt and o/exTrxf plants under light or dark conditions (Figure 8c). These findings suggest that the overexpression of NTRC in tobacco plants leads to a downregulation of a putative DBE during the night, which could explain, at least to some extent, the starch increase that was seen in these plants.

### 2.7. NTRC and Trx f Overexpression Differentially Alters the Net Starch Synthesis and Degradation

**Figure 9:**
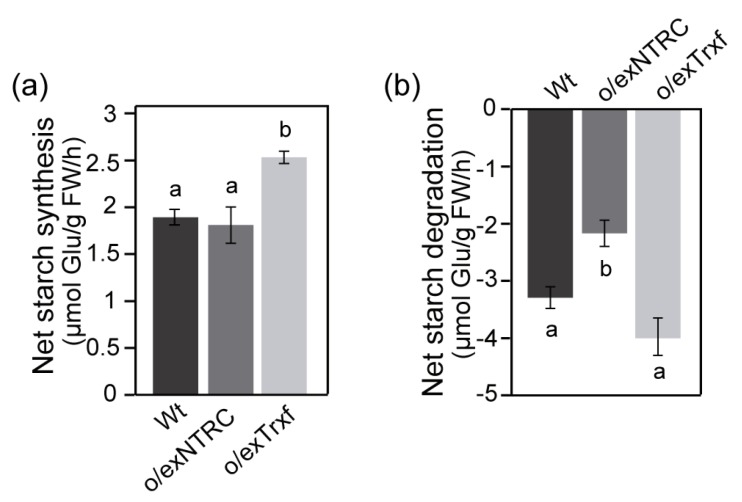
Net starch synthesis (**a**) and degradation (**b**) in homoplasmic plants overexpressing NTRC or Trx f (T_1_ generation). Pairs of samples were harvested from equivalent leaves of Wt, o/exNTRC, and o/exTrxf plants at the end of the light (16 h) and dark (8 h) periods. The net starch synthesis and degradation were calculated as reported in “Material and methods”. The results are the mean ± SE for seven individual plants. Different letters above each bar indicate significant differences (*p* < 0.05, ANOVA).

Net starch synthesis and degradation were determined to further investigate the differential role of NTRC and Trx f overexpression in promoting starch accumulation in tobacco transplastomic plants. Our results showed that, during the light period, NTRC overexpression in the chloroplasts did not alter the net starch synthesis when compared to Wt (≈1.8 µmol glucose.g FW^−1^ h^−1^), whereas it was greatly increased by about 30% in o/exTrxf leaves (Figure 9a). However, contradicting results were found when nocturnal net starch degradation was analyzed (Figure 9b). After 8 h of darkness, only o/exNTRC plants exhibited a significant reduction of about 40% in the net starch degradation when compared to o/exTrxf and Wt plants. Overall, these results indicate that the overexpression of NTRC or Trx f in the chloroplast leads to an increased leaf starch content, although different mechanisms seem to be governing starch metabolism in each transplastomic line.

## 3. Discussion

Chloroplasts contain a rich diversity of Trxs, whose reduction is dependent on Fd reduced by the photosynthetic electron transport chain, and ultimately on light. Meanwhile, NTRC forms a complete Trx system in a single polypeptide that relies on NADPH and, thus, might also be operative during the night. Trxs and NTRC are both reported to control multiple plant processes, including the biosynthesis of starch [24,54]. Previous studies led to the view that these two systems may have non-overlapping functions in plants [55]. In this work, we have explored the specificity of NTRC and Trx f in the regulation of starch metabolism by using overexpression and reverse genetic approaches.

### 3.1. Phenotype of N. Tabacum and N. Benthamiana Plants with Altered NTRC Protein Levels

We show that the overexpression of a fully functional NTRC protein in chloroplasts significantly increases the transitory starch content in tobacco leaves (Figure 4a) and partially alters its phenotype (slight growth delay and lower chlorophyll content than Wt) during the younger growth stages (Figure 2). However, the o/exNTRC tobacco plants recover the Wt phenotype in their adult stage (Table S1). NTRC has been unveiled as a redox regulatory system in chloroplasts that reduces target proteins at the expense of NADPH [10]. Thus, the recovery of the Wt phenotype may indicate that the overexpressed NTRC in tobacco plants competes for NADPH with the accelerated chloroplast metabolism of growing tissues, but not in adult stages. In agreement with our results, the overexpression of NTRC in Arabidopsis was previously shown to increase the starch content in illuminated leaves, which also displayed chlorophyll reduction [56]. The importance of redox regulatory mechanisms in the maintenance of well-adjusted tetrapyrrole biosynthesis during plant development has been broadly demonstrated, with NTRC being a key player in this regulation [57]. Hence, NTRC overexpression might alter the redox status of the chloroplast in young plants, thereby affecting chlorophyll synthesis.

In contrast, we found that *N. benthamiana* silenced VIGS-NTRC plants showed no apparent phenotypic changes when compared with non-silencing control VIGS-GUS plants, and the two sets of plants accumulated similar amounts of transitory starch (Figure 3a and Figure 4b). Likewise, it was determined that Arabidopsis *ntrc* mutant plants that were grown at low irradiance did not show differences when compared to Wt in either plant growth or the patterns and rates of starch accumulation [34]. On the contrary, an attenuation of starch content has widely been reported in *ntrc* Arabidopsis single mutants [28,29,30,56,58,59], which exhibit impaired growth phenotype. However, it should be noted that most of these studies were carried out at light intensities exceeding 125 µmol m^−2^ s^−1^, where the plants grew under photo-oxidative stress conditions. In that case, NTRC would play an important role in protecting plants against such stresses [34]. Recently, the impaired growth of an Arabidopsis *ntrc* mutant has been associated with an increased electron flow from the Trx pool to 2-Cys Prx (involved in reducing H_2_O_2_) that might indirectly downregulate the Calvin–Benson cycle [60]. Thus, the lack of phenotype in VIGS-NTRC silenced plants may be explained by the low-irradiance growing conditions, where limited H_2_O_2_ production is presumed. Under such conditions, the influence of NTRC silencing on starch metabolism can therefore be better analyzed.

### 3.2. Enhanced Starch Content in o/exNTRC Leaves as a Consequence of Impaired Starch Metabolism at Night

The increased amount of transitory starch that was seen in o/exNTRC leaves was not accompanied by either upregulation of SS activity (Figure 5a) or redox-activation of AGPase during the day (Figure 6a). Accordingly, no differences were found in the net starch synthesis that was calculated for o/exNTRC plants when compared to the Wt (Figure 9a). There is general agreement regarding the role of NTRC in regulating starch synthesis via AGPase reduction [28,30,58]. In this work, the o/exNTRC plants showed a significant increase of AGPB monomerization, but only during the night (Figure 6a). It may be that, in illuminated chloroplasts, overexpressed NTRC competes with the active chloroplast metabolism for photosynthetically-generated NADPH, while the availability of NADPH generated via the OPPP in the dark would supply more electrons to the NTRC system. We analysed the reduction pattern of a well-known NTRC target (2-Cys Prx) in o/exNTRC plants, and demonstrated that it is also more efficiently reduced at night, with up to a four-fold increase in 2-Cys-Prx reduction s compared to light conditions to investigate this hypothesis (Figure S3). In agreement with these results, it was previously shown that 2-Cys Prx was more reduced in Arabidopsis plants overexpressing NTRC under dark conditions than in Wt plants [61]. All in all, the present findings point to a higher reductive activation of both AGPase and 2-Cys Prx by overexpressed NTRC at night, when NADPH availability is apparently higher. Interestingly, during the night, increased AGPB monomerization in o/exNTRC plants converged with a reduction in starch turnover (Figure 9b). Thus, AGPase redox-activation in darkness could account for the modified starch turnover in the o/exNTRC leaves by supporting starch synthesis. Similarly, it was previously suggested that Arabidopsis lines expressing a mutagenized and permanently active AGPase accumulated more leaf starch, which was probably due to a slow starch turnover during the night [33]. When VIGS-NTRC silenced plants (grown under non-stressed conditions) were analyzed for AGPase reduction, the proportion of fully reduced AGPB in the light was significantly decreased when compared to Wt (Figure 6b), which is in agreement with the previously reported results [28,30]. However, contrary to these works, no changes in the transitory starch accumulation occurred in VIGS-NTRC silenced plants (Figure 4b). Our findings suggest that NTRC is involved in AGPase redox-regulation, although this does not appear to have a direct impact on starch biosynthesis during the day, at least under the assayed conditions. Other authors have also questioned the role of AGPase redox-activation in starch biosynthesis [34,35], and starch synthesis stimulation in vivo has been demonstrated, independent of such reductive activation [48,62].

In recent years, additional candidates have been added to the list of reductively activated starch metabolizing enzymes, some of which are related to starch degradation [24,36]. Besides the Fd/Trx system, which is presumed to upregulate starch degradation during stress conditions or in guard cells under light conditions [39,46], NTRC could also provide reductive regulation of these enzymes in the night. Indeed, BAM1 from Arabidopsis has been described to be partially reduced by NTRC in vitro [39]. Therefore, a role for the overexpressed NTRC in the redox control of starch degradative enzymes in tobacco leaves during the night cannot be ruled out. However, in the present work, we show that the activity of AMY, BAM, and LDA enzymes in o/exNTRC or VIGS-NTRC plants did not differ from that of the control plants (Figure 7), which suggested that the level of NTRC accumulated in chloroplasts does not affect the overall amylase and LDA activities in vivo under the assayed conditions. Interestingly, zymographic analysis that was performed with o/exNTRC extracts revealed a decrease in the activity of a hydrolytic enzyme in dark conditions when compared to Wt (Figure 8b). Therefore, our results rather argue for an amylolytic enzyme deactivation in o/exNTRC plants, which is in agreement with the reduction of its net starch degradation (Figure 9b). Although Trx-mediated deactivation of other chloroplastic enzymes, like glucose-6-phosphate dehydrogenase, has previously been described [63,64], this may be the first evidence for a starch-degrading enzyme. The mobility of this downregulated degradative enzyme in the zymogram, which also seems to be deactivated in Wt tobacco extracts that were incubated with DTT (Figure 8a), indicates that it might correspond with a putative DBE [53]. Two DBEs involved in starch breakdown has been reported: ISA3 and LDA. LDA was previously described as a redox-sensitive enzyme, although it seems to be more active under reducing conditions [36,65,66]. However, as stated before, no differences in LDA activity were seen between the NTRC transgenic plants and their respective controls (Figure 7c,f). On the other hand, a small but significant activation of the Arabidopsis ISA3 activity was observed under reducing conditions [36]. In vivo analysis of DBE mutants shows that the Arabidopsis *isa3* mutants accumulate more leaf starch and have a slower rate of starch breakdown than Wt plants [67,68], a similar phenotype as o/exNTRC plants. All in all, these findings suggest a possible downregulation of ISA3 activity in o/exNTRC tobacco plants being exerted by the overexpressed NTRC, taking that no changes were found in *ISA3* gene expression in these transplastomic plants (Figure S1). This regulation could explain, at least in part, the increased starch content and reduced starch turnover found in these plants. Further investigations are required to shed light on this matter.

Finally, it should be noted that qualitative zymogram analysis also showed a putative AMY isoform [53] that appears to be activated in Wt extracts that were treated with DTT as reducing agent (Figure 8a). This enzyme could be an ortholog of the Arabidopsis plastid-localised AMY3, being the unique redox-regulated AMY isoform that was reported in chloroplasts [36,38]. However, our results showed that neither NTRC nor Trx f overexpression appeared to alter the redox status of this AMY isoform in tobacco leaf extracts (Figure 8b,c).

### 3.3. Starch Synthase as the Main Determinant of Starch Accumulation in Trx f Transgenic Plants

We previously demonstrated that the overexpression of Trx f in tobacco chloroplasts has a positive effect on transitory starch accumulation and leaf biomass production [48,69,70]. Consistent with this, here we show a decrease in starch content in the corresponding Trx f silenced plants, which did not display any visible phenotype (Figure 3a and Figure 4b). Previous work also showed that Arabidopsis *trxf1* mutants led to a decrease in starch content without altering the plant phenotype [30,32]. However, double *trxf1-f2* mutants that also accumulated less transitory starch showed some phenotypic defects [29,31]. Some of these studies with Arabidopsis mutants that were attributed the decrease in transitory starch to the AGPase deactivation [30,32]. However, we have previously demonstrated that the redox state of AGPase was not altered in Trx f-overexpressing tobacco plants [48], and neither was it in VIGS-Trxf plants (Figure S2).

Here, we demonstrated that SS activity in o/exTrxf plants was higher than in Wt plants (Figure 5a), while VIGS-Trxf plants showed decreased SS capacity (Figure 5b). Moreover, the SS transcript levels were unaltered in o/exTrxf plants when compared to Wt (Figure S1), which pointed towards a posttranslational regulation of SSs by Trx f. It must be noted that there are five classes of SS in higher plants (SSI, SSII, SSIII, SSIV, and GBSS) that are involved in starch biosynthesis. Based on mutant phenotypes, each SS class appears to have a distinct role during amylopectin synthesis, although their relative contribution varies in different tissues and among species [22]. SSI constitutes the major soluble SS and it is a major determinant for the synthesis of amylopectin in Arabidopsis leaves [71]. Moreover, based on the crystal structure of barley SSI, it was proposed that a disulfide bridge between two Cys can be formed [72]. Interestingly, the ability of Arabidopsis SSI to be redox-activated was confirmed, with Trx f being the most effective activator in vitro [37]. Supporting this idea, Arabidopsis plants overexpressing Trx f from different species displayed increased SS expression and activity [73,74]. Taken together, our results also support the view of a specific role of Trx f in the redox-regulation of starch synthases in tobacco plants. Accordingly, the net starch synthesis was increased in o/exTrxf plants, while the net starch degradation remained unaltered (Figure 9), which underpinned the idea that Trx f seems to be mainly involved in starch biosynthesis modulation.

## 4. Materials and Methods

### 4.1. Plant Material and Growth Conditions

Arabidopsis *ntrc* coding sequence (GenBanK: NM_129731), excluding the putative transit peptide, was amplified by PCR while using the primers described in Table S2 to generate o/exNTRC plants. The amplified *ntrc* sequence, which included a 6xHis tag to facilitate protein purification, was cloned into a pKS intermediate vector (Stratagene, La Jolla, CA, USA) for fusion to the promoter and 5′UTR of the tobacco *psbA* gene. Finally, the NTRC expression cassette was introduced into the chloroplast transformation vector pAF [75] to generate pAF-NTRC. Gold microprojectiles that were coated with pAF-NTRC vector were bombarded into *Nicotiana tabacum* (Petite Havana SR1) in vitro-grown leaves, as described previously [51]. Two rounds of selection and shoot development on RMOP medium containing 500 mg/L spectinomycin were performed. Regenerated plants were transplanted and grown in a phytotron (16 h light/8 h dark, 150 µmol m^−2^ s^−1^ and 28 °C) for homoplasmy confirmation and seed production. Untransformed tobacco plants (Wt) and previously generated tobacco plants overexpressing the *NtTrxf* sequence from the chloroplast genome [48] (referred to as o/exTrxf) were also used in this study.

Silenced plants, in which the expression of *ntrc* or *Trxf* genes was strongly reduced, were also generated in this study (referred to as VIGS-NTRC and VIGS-Trxf). To obtain these plants, tobacco rattle virus-based VIGS was applied in *N. benthamiana* [76]. A database search (*Nicotiana benthamiana* Genome and Transcriptome Sequencing Consortium: http://benthgenome.com) identified the tobacco orthologous *ntrc* and *Trxf* genes in *N. benthamiana* by BLAST homology. In both cases, all the found sequences had high similarity and a conserved region was used to specifically silence each gene. *ntrc* and *Trxf* conserved fragments were amplified from RT-PCR-generated *N. benthamiana* cDNA while using the primers described in Table S2 and cloned into the pTRV2 vector. pTRV1, which contains the replication and movement viral genes, and pTRV2-NTRC or -Trxf derivatives were introduced into *Agrobacterium tumefaciens* GV3101 and co-agroinfiltrated into 3–4-week-old *N. benthamiana* plants according to a standard protocol [77,78]. The non-silencing TRV control, containing a 396-bp fragment of the β-glucuronidase gene (GUS), was used as described previously [79]. The silencing of the endogenous phytoene desaturase (*PDS*) gene, which causes photobleaching, was used as a positive control for VIGS efficiency (VIGS-PDS). Silenced plants were grown in a phytotron in 16 h light/8 h dark light regime at 80 µmol m^−2^ s^−1^ and 24 °C. The plants were grown for three weeks prior to analysis, allowing for the post-infiltration development of at least 2–3 full leaves.

### 4.2. DNA, RNA and Protein Analysis

A Southern blot analysis was performed to analyze homoplasmy in the T_1_ generation of plants overexpressing NTRC. Leaf discs from o/exNTRC and wild-type (Wt) tobacco plants were finely powdered in liquid nitrogen. Total plant DNA was extracted by using the cetyltrimethylammonium bromide (CTAB) procedure [80]; 10 µg were digested with *Bgl*II, separated on a 0.8% (*w/v*) agarose gel, transferred to a nylon membrane, and then hybridized with a 0.8 kb probe homologous to the flanking sequences. Probe labelling and hybridization were performed while using the DIG High Prime DNA Labelling and Detection Starter Kit II (Roche, Mannheim, Germany).

Analysis of *ntrc* and *Trxf* mRNA levels was carried out in VIGS-NTRC and VIGS-Trxf plants. Total RNA from silenced leaf tissues was extracted using Trizol^®^ Reagent (Thermo Fisher Scientific, Waltham, CA, USA), while following the manufacturer’s protocol. The generation of cDNA and RT-qPCR analysis were performed, as previously described [81]. The efficiency of the primers used in RT-qPCR was no lower than 98%. To normalize the mRNA levels of target genes between samples, the relative actin mRNA levels were determined using actin-specific primers (Table S2) and a relative quantification method [82].

Protein expression was analysed in the T_1_ generation of o/exNTRC homoplasmic plants, as well as in VIGS plants. The total protein was extracted in Laemmli buffer (0.5 M Tris–HCl pH 6.5, 4% SDS, 20% glycerol, and 10% β-mercaptoethanol) and quantified while using the DC Protein Assay (Bio-Rad, Hercules, CA, USA) with bovine serum albumin as a standard. The proteins were electrophoresed on a 10% or 15% SDS-polyacrylamide gels for NTRC and Trx f, respectively, transferred to a PVDF membrane and immunoblotted with specific antibodies: 1:750 dilution for NTRC [10] and 1:5000 for Trx f [51]. A peroxidase-conjugated goat anti-rabbit antibody (1:10,000; Sigma-Aldrich, St Louis, MO, USA) was used as secondary antibody. Detection was performed while using the ECL Prime detection system (GE Healthcare, Buckinghamshire, UK).

### 4.3. Protein Purification and Activity Assays

Fully expanded leaves of the T_1_ generation of o/exNTRC homoplasmic plants were ground in liquid nitrogen and homogenized 1:5 (*w/v*) in protein extraction buffer [20 mM sodium phosphate pH 7.4, 500 mM NaCl, 0.1% (*v/v*) Triton X-100, including a cocktail of protease inhibitors from Roche (Mannheim, Germany)]. The homogenate was incubated on ice for 45 min and cell debris was pelleted by centrifugation (20,000× *g*, 20 min, 4 °C). Overexpressed NTRC was purified from plant protein extracts by affinity chromatography on a Ni-NTA column (Qiagen, Hilden, Germany). The protein content of eluates was measured with the DC Protein Assay (Bio-Rad, Hercules, CA, USA) and the purity of NTRC was checked by SDS-PAGE and Coomassie Blue staining. The Trx activity of the purified NTRC protein (2, 4, or 8 µM) was determined according to the DTT-dependent insulin reduction assay [2], as described previously [83]. The NTR activity was determined by the reduction of 5,5′-dithiobis(2-nitrobenzoic acid) (DTNB) [10].

### 4.4. Starch Determination

The starch determination was performed while using an amyloglucosidase-based test kit (R-Biopharm AG, Darmstadt, Germany), according to the manufacturer’s instructions. Starch content was analysed in fully-expanded young leaves of the T_1_ generation of o/exNTRC and o/exTrxf homoplasmic plants harvested just before the inflorescence emission (seven-week-old plants) at the end of the light (16 h) and dark (8 h) periods. Starch was also analyzed in silenced plants (VIGS-NTRC and VIGS-Trxf) while using fully expanded leaves that were collected three weeks after infection.

For net starch synthesis and degradation determination, starch content at the end of the dark (8 h) and the light (16 h) periods were measured and the slopes between both of those times were calculated. For each determination, the paired samples were collected at the same position of the leaf blade at both sides of the central vein.

### 4.5. Enzyme Activities Associated with Starch Synthesis and Degradation

Leaves from tobacco Wt, T_1_ generation of o/exNTRC and o/exTrxf homoplasmic plants, and *N. benthamiana* VIGS-GUS, VIGS-NTRC, and VIGS-Trxf plants were harvested after 8 h illumination or 4 h darkness and immediately frozen and ground in liquid nitrogen.

Enzymatic analysis of soluble starch synthase activity (not specific for any particular isoform) was carried out in protein extracts from leaves sampled in the light according to [84]. Briefly, the leaves were homogenized on 100 mM Tricine-NaOH (pH 8.0), 8 mM MgCl_2_, 2 mM EDTA, 12.5% (*v/v*) glycerol, and 5% (*w/v*) insoluble polyvinylpyrrolidone-40, and then centrifuged at 10,000× *g* for 5 min to obtain soluble protein in the resulted supernatant. The extract was incubated for 20 min at 30 °C in a buffer containing 50 mM HEPES-NaOH (pH 7.4), 1.6 mM ADPglucose, and 2.5 mg/mL amylopectin. The enzyme was inactivated by 30 s at 99 °C. Subsequently, the mixture was incubated with a solution of 50 mM HEPES-NaOH (pH 7.4), 4mM PEP, 200 mM KC1, 10 mM MgCl_2_, and 12 units/mL of pyruvate kinase, and then incubated for 30 min at 30 °C. The mixture was then boiled for 30 s and centrifuged at 10,000× *g* for 5 min. The supernatant was mixed with a solution of 50 mM HEPES-NaOH (pH 7.4), 10 mM glucose, 20 mM MgCl_2_, and 2 mM NADP. The enzymic activity was measured as the increase in absorbance of 340 nm after the addition of hexokinase (4.5 units/mL) and glucose-6-phosphate dehydrogenase (1 unit/mL). One unit of activity was defined as the amount of enzyme causing an increase of one unit per min in absorbance at 340 nm.

The activities of AMY and BAM were measured in leaves harvested in the dark while using the Ceralpha^®^ and Betamyl-3^®^ assay kits (Megazyme, Bray, Ireland). Both of the assays are highly specific and selective for each enzyme and avoid interferences from DBEs. For LDA assay, the PullG6 Method (Megazyme, Bray, Ireland) was used according to the manufacturer’s instructions in leaves that were harvested in dark conditions.

### 4.6. AGPB Redox Status

The AGPase redox status was determined by immunoblotting, analysing the degree of AGPB monomerization in leaf samples that were collected at the end of the light (16 h) and dark (8 h) periods. Protein extraction was performed, as previously described [27]. Proteins from 1 mg of fresh weight were subjected to 10% non-reducing SDS-PAGE, transferred to nitrocellulose membrane, and then probed with a specific AGPase antibody (Agrisera AB, Vännäs, Sweden) at a dilution of 1:1000. A peroxidase-conjugated goat anti-rabbit antibody (Sigma-Aldrich, Saint Louis, MO, USA) at a 1:10,000 dilution was used as secondary antibody.

### 4.7. Zymograms of Starch Hydrolytic Activities

The leaf samples (300 mg) harvested after 8 h light or 4 h dark were ground in liquid nitrogen, homogenized in 1 mL of soluble protein extraction buffer (50 mM Hepes pH 7.5, 2 mM EDTA, and 10% glycerol), and then incubated for 5 min on ice. The supernatant was obtained after 10 min of centrifugation at 14,000× *g* at 4 °C and soluble protein concentration was measured by Bradford assay (Bio-Rad, Hercules, CA, USA) while using bovine serum albumin as a standard. Protein extracts (35 µg) were incubated in the presence or absence of 40 mM DTT or 200 µM CuCl_2_ for 2 h on ice in the dark and loaded on native PAGE gel (7.5% acrylamide) containing 0.2% (*w/v*) potato amylopectin (Sigma-Aldrich, Saint Louis, MO, USA) as substrate in the separating gel. After migration (under native condition for 4 h at 4 °C at 15 V.cm^−1^), the gels were incubated overnight at room temperature in 100 mM Tris-HCl pH 7, 1 mM MgCl_2_, and 1 mM CaCl_2_ containing buffer. Activities were revealed by iodine staining (Lugol solution; Sigma-Aldrich, Saint Louis, MO, USA).

## Supplementary Materials

The following are available online at https://www.mdpi.com/2223-7747/8/12/543/s1, Figure S1: RT-qPCR analysis of *SS1*, *SS3*, *ISA3* and *LDA* expression in tobacco Wt, o/exTrxf and o/exNTRC homoplasmic plants (T_1_ generation), Figure S2: Redox-activation ofAGPase in VIGS-Trxf *N. benthamiana* leaves, Figure S3: Redox status of 2-Cys Prx in the T_1_ generation of o/exNTRC homoplasmic plants, Table S1: Phenotypic characterization of the T_1_ generation of o/exNTRC homoplasmic plants grown in phytotron under standard conditions at different developmental stages, Table S2: List of primers used in this work.
