# Supplementary material for: NTRC and Thioredoxin f Overexpression Differentially Induces Starch Accumulation in Tobacco Leaves
Source: Plants (Basel). 2019 Nov 26;8(12):543. doi: 10.3390/plants8120543 (PMC6963466; doi:10.3390/plants8120543)
Supplement: Supplementary file 1 [file plants-08-00543-s001.pdf]

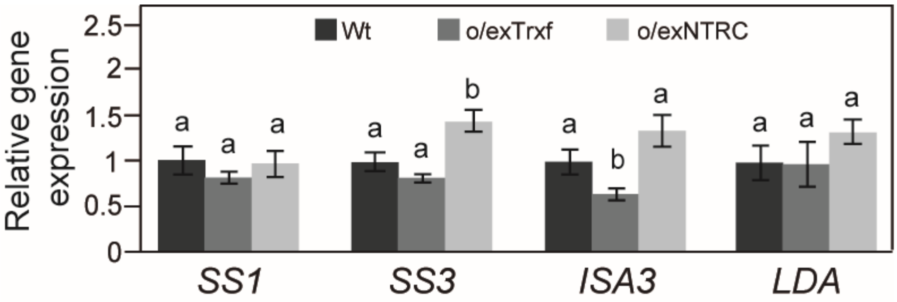

Figure S1. RT-qPCR analysis of SS1, SS3, ISA3 and LDA expression in tobacco Wt, o/exTrxf and o/exNTRC homoplasmic plants (T_1 generation). Graphical representation of relative expression levels normalized to 16S rRNA expression that shows the fold change as the mean of the different biological repeats (n= 3). Error bars represent SE.

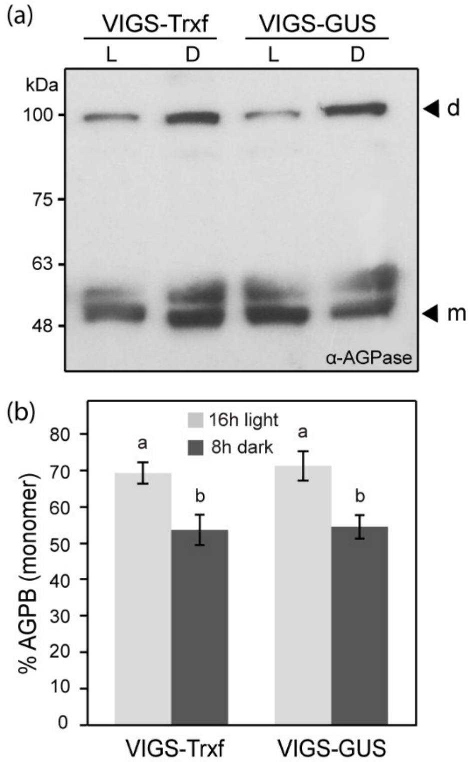

Figure S2. Redox activation of AGPase in VIGS-Trxf N. benthamiana leaves. Redox status of AGPase of mature leaves from Wt and VIGS-Trxf plants grown in phytptron sampled at the end of the light (L; 80 µmol.m^{-2}.s^{-1}) and dark (D) periods. (a) A representative non-reducing western blot is shown. Specific AGPB antibody was used. m: monomer; d: dimer. (b) Quantification of AGPB monomerisation by western blot analysis shown as the percentage of the 50 kDa monomer relative to the total amount of AGPB. Results are the mean ± SE of four individual plants. Different letters above the bars represent significant differences (P< 0.05, ANOVA).

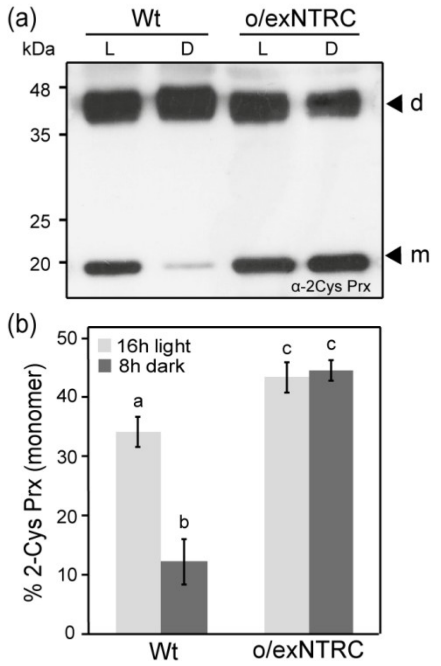

Figure S3. Redox status of 2-Cys Prx in the T_1 generation of o/exNTRC homoplasmic plants. Redox activation of 2-Cys Prx in leaves from 7-week-old o/exNTRC tobacco plants grown in phytotron and sampled at the end of the light (L) and dark (D) periods. (a) A representative non-reducing western blot of 2-Cys Prx in leaves of Wt and o/exNTRC plants is shown. m: monomer; d: dimer. (b) Quantification of 2-Cys Prx monomerization shown as the percentage of the 20 kDa monomer relative to the total amount of 2-Cys Prx. Results are the mean ± SE for five plants. Different letters above each bar indicate significant differences ($P < 0.05$, ANOVA).

Table S1. Phenotypic characterization of the T_1 generation of o/exNTRC homoplasmic plants grown in phytotron under standard conditions at different developmental stages. Values are the mean ± SE for 6 individual plants. Statistical significance compared to Wt plants is indicated by asterisks ($P < 0.05$, Student's t-test).

	**Wt**	**o/exNTRC**
Flowering stage		
Height (cm)	82.5 ± 4.2	70.0 ± 2.7*
Chlorophyll content (SPAD)	32.8 ± 1.5	30.5 ± 0.9
Maturity		
Height(cm)	87.0 ± 4.5	86.7 ± 4.5
Chlorophyll content (SPAD)	26.5 ± 1.9	26.8 ± 0.9

Table S2. List of primers used in these work.

**Primers used for NTRC amplification (5'-3')[a,b]**		
NTRC_for	ccatgggt**catcaccatcaccatcac**GGATCCTCTTCTTCAGAGGCGAG	
NTRC_rev	gcggccgcTTATTTATTGGCCTCAA	
**Primers used for VIGS vector construction**		**Amplicon size (bp)**
pTRV2-NTRC_for	AGTgaattcCTGATACTCAGGAAGAATC	
pTRV2-NTRC_rev	CTAggatccACTGTACACATGGAGTAC	585
pTRV2-trxf_for	AGTgaattcGTTGCAGGAGATTATGGC	
pTRV2-trxf_rev	CTAggatccCTTTGGAGCAATCAC	228
**Primers used for RT-qPCR analysis**		**Amplicon size (bp)**
Actin_for	CAGCAACTGGGATGATATGG	
Actin_rev	GGCGCTTCAGTTAAGAGGAC	99
NTRC_for	TGACTTAATGGATAGGATGAG	
NTRC_rev	GTTCACTACTTTGGATAGTAA	116
trx f_for	CAAGTAAACGGCGTATCATT	
trx f_rev	CAGACCACCGATTGCTTGCT	81
16S rRNA_for	CTTTTTAAGTCCGCCGTCAAA	
16S rRNA_rev	TCTTTCCGATCTCTACGCATTTC	127
SS1_for	GCTGAAGCAGCTCCGTATTC	
SS1_rev	GAGACGACCATTACACGATG	101
SS3_for	AAAACCCGATGAAGATGTCG	
SS3_rev	GCGCCAGTCATTAAAAGCTC	100
ISA3_for	AGTGCTTCTCGGGAGTTCAA	
ISA3_rev	CGGGTTTTTGTCATCAGCTT	111
LDA_for	GTGAAGGCAAAGTCATTGCT	
LDA_rev	TGTTTGCCACTTCACCAAAA	109

[a] Restriction sites are indicated in lowercase

[b] 6xHis tag is indicated in bold.
